# 10040 Proactive and responsive COVID-19 multidisciplinary research support through the University of Minnesota’s Clinical Research Support Center

**DOI:** 10.1017/cts.2021.676

**Published:** 2021-03-30

**Authors:** Brian L. Odlaug, Francoise Crevel, Nicole Tosun, Ryan Lee, Carrie McKenzie, Melena Bellin, Brenda Prich, Daniel Weisdorf

**Affiliations:** 1 Clinical and Translational Science Institute, University of Minnesota, Minneapolis, MN, USA; 2 Department of Pediatrics and Surgery, University of Minnesota Medical Center, Minneapolis, MN, USA; 3 Division of Hematology, Oncology, and Transplantation, Department of Medicine, University of Minnesota, Minneapolis, MN, USA

## Abstract

ABSTRACT IMPACT: In a global pandemic where data development and dissemination are integral to combating the disease, the Clinical Research Support Center at the University of Minnesota provides a model of comprehensive virtual support, helping to attain and disseminate novel research on COVID-19, its individual and community impact, and treatment initiatives/outcomes. OBJECTIVES/GOALS: The pandemic created massive disruption to the conduct of clinical research with an unprecedented reorientation towards COVID-19. In this fast-paced environment, the Clinical Research Support Center (CRSC) rapidly developed innovative means of supporting diverse research initiatives. METHODS/STUDY POPULATION: The CRSC rapidly transitioned into a virtual environment and developed tools for the clinical research community to enhance remote clinical trial start up. This includes supporting remote consent, eBinders, COVID-19 research training for clinical staff, and easier identification of potential participants for COVID-19 studies; all through virtual support. Support provided research teams guidance on study protocols, regulatory requirements, informatics, biostatistics, financial management, recruitment strategies to support critical, urgent COVID-19 research. We outline proactive examples of how the CRSC now provides support to research teams through the pandemic. RESULTS/ANTICIPATED RESULTS: From March-November 2020, 116 COVID-19 projects received virtual support from the CRSC for COVID-19 research: disease understanding (n=27), treatment (n=23), pandemic impact (n=20), clinical care innovation (n=18), disease control and surveillance (n=10), prevention (n=9), detection (n=5), and impact on minorities (n=4). The diversity of these studies demonstrates the demand for and benefit from multidisciplinary expertise supporting study design and implementation. Through successful articulation and acceleration of research activities, the CRSC met the need for speed and rapidly adapted to new challenges created by the pandemic. DISCUSSION/SIGNIFICANCE OF FINDINGS: In a global pandemic where rapidly changing barriers to research is ongoing, through multidisciplinary efforts, the CRSC continues to provide comprehensive, virtual support to attain and disseminate novel research on COVID-19, its individual and community impact, and treatment initiatives/outcomes.

